# A Trace C_2_H_2_ Sensor Based on an Absorption Spectrum Technique Using a Mid-Infrared Interband Cascade Laser

**DOI:** 10.3390/mi9100530

**Published:** 2018-10-19

**Authors:** Ye Mu, Tianli Hu, He Gong, Ruiwen Ni, Shijun Li

**Affiliations:** College of Information Technology, Jilin Agricultural University, Changchun 130018, China; hutianli@jlau.edu.cn (T.H.); gonghe@jlau.edu.cn (H.G.); niruiwenjlau@163.com (R.N.); lishijun@jlau.edu.cn (S.L.)

**Keywords:** trace C_2_H_2_ detection, mid-infrared spectrum, interband cascade laser, tunable semiconductor laser absorption spectroscopy, wavelength modulation technology, minimum detection limit

## Abstract

In this study, tunable diode laser absorption spectroscopy (TDLAS) combined with wavelength modulation spectroscopy (WMS) was used to develop a trace C_2_H_2_ sensor based on the principle of gas absorption spectroscopy. The core of this sensor is an interband cascade laser that releases wavelength locks to the best absorption line of C_2_H_2_ at 3305 cm^−1^ (3026 nm) using a driving current and a working temperature control. As the detected result was influenced by 1/*f* noise caused by the laser or external environmental factors, the TDLAS-WMS technology was used to suppress the 1/*f* noise effectively, to obtain a better minimum detection limit (MDL) performance. The experimental results using C_2_H_2_ gas with five different concentrations show a good linear relationship between the peak value of the second harmonic signal and the gas concentration, with a linearity of 0.9987 and detection accuracy of 0.4%. In total, 1 ppmv of C_2_H_2_ gas sample was used for a 2 h observation experiment. The data show that the MDL is low as 1 ppbv at an integration time of 63 s. In addition, the sensor can be realized by changing the wavelength of the laser to detect a variety of gases, which shows the flexibility and practicability of the proposed sensor.

## 1. Introduction

Acetylene (C_2_H_2_) is one of the most important industrial gases used in industrial production, and it easily decomposes, burns, and explodes. Compared with other inflammable and explosive gases, C_2_H_2_ has a lower explosion limit. In recent years, there have been many reports of C_2_H_2_ explosion, which has brought great loss to people’s safety and social production. Therefore, developing a sensor to monitor C_2_H_2_ with high accuracy and sensitivity in real time is highly important.

The absorption intensity of gas molecules in the mid-infrared band is nearly three orders of magnitude stronger than that in the visible or near-infrared spectrum band [1]. Under the same measurement conditions, the signal intensity obtained via gas concentration detection in the mid-infrared band is several times higher than that obtained in the visible and near infrared bands, which behaves with better detection accuracy and minimum detection limit (MDL) in the ppbv level [2,3,4,5,6,7]. Therefore, high sensitivity detection of essential C_2_H_2_ gas in chemical production using the spectrum absorption lines in mid-infrared band is an effective detection method [8,9].

Interband cascade lasers (ICLs), combined with tunable diode laser absorption spectroscopy (TDLAS)-wavelength modulation spectroscopy (WMS), are used to detect trace C_2_H_2_. The emitting light of ICL with a center wavelength of 3026 nm is tuned by the driving current and working temperature, which sweep the best absorption lines of C_2_H_2_. According to the Beer–Lambert law, the C_2_H_2_ concentration is deduced by measuring the attenuation of laser intensity. The proposed C_2_H_2_ sensor’s detection accuracy is 0.4%, the MDL is as low as 1 ppbv, and the stability is better than 1.776 × 10^−2^.

## 2. Detection Principle of C_2_H_2_ Using Absorption Spectroscopy

### 2.1. Selection of C_2_H_2_ Absorption Line

**Figure 1:**
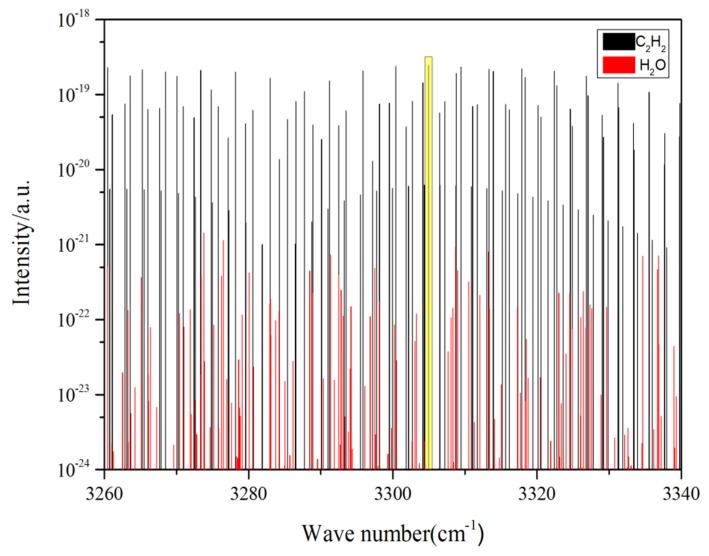
Absorption lines of C_2_H_2_ and H_2_O in the range of 3290–3320 cm^−1^.

The absorption spectrum refers to the fraction of incident radiation that is absorbed by the material over a range of frequencies. The absorption spectrum is primarily determined by the molecular composition of the material [10,11]. Except for diatomic and inert gases without polar symmetrical structure, each material has its own characteristic absorption spectrum. Thus, this characteristic can be used to identify gas molecules [12,13]. According to the high-resolution transmission molecular absorption database (HITRAN) database [14], the mid-infrared band absorption spectrum of C_2_H_2_ was searched to determine the absorption capacity of C_2_H_2_ at different wavelengths. As shown in Figure 1, C_2_H_2_ has a significant absorption spectrum line in the range of 3290–3320 cm^−1^ in comparison to H_2_O, which may be present in significant amounts in the gas mixture.

As shown in Figure 1, the x coordinate is the wave number (reciprocal centimeters, cm^−1^), the y coordinate is the absorption intensity (a.u.), the black lines represent the C_2_H_2_ gas absorption lines, and blue lines are H_2_O absorption lines. To improve the accuracy of the measured results, a C_2_H_2_ absorption line centered at 3305 cm^−1^ (3026 nm) with an absorption magnitude of 10^−19^ was selected as the optimum C_2_H_2_ target line. All of the H_2_O absorption lines (the two closest lines are located at 3303 cm^−1^ and 3308 cm^−1^) under an absolute humidity of 2% did not interfere with the selected C_2_H_2_ line at 3305 cm^−1^, since they were ∼2 to 3 cm^−1^ away. With a higher relative humidity, the dryers could be used to lower the H_2_O concentration and thereby reduce the absolute humidity to an acceptable level, e.g., below 2%. In that case, the sensor could operate normally.

### 2.2. Derivation of TDLAS-WMS

TDLAS is based on the principle of the Beer–Lambert law, which states that absorbance is proportional to the concentrations of the attenuating species in the material sample [15,16]. It can be expressed as follows:

(1) $$I_{1}=I_{0}e^{-\alpha (v)PCL}$$

where *I*_0_ is the emitting light intensity of the laser, *I*_1_ is the light intensity after passing the measured gas, *L* is the effective length of absorption optical path, *P* is the pressure in the cell, *C* is the gas concentration, and α(v) is the molecular absorption coefficient. Then, α(v) can be expressed as follows:

(2) $$\alpha (v)=T(t)\times g(v-v^{\prime })\times N$$

where *T*(*t*) is the absorption intensity of gas at time point *t*, $g(v-v^{\prime })$ is a linear function of the measured gas, $v^{\prime }$ is the initial frequency of energy level transition of the gas molecule, and *N* is the number of molecules per volume. To improve the MDL performance of the C_2_H_2_ sensor, TDLAS-WMS was adopted to eliminate the 1/*f* noise caused by ICL or external environmental disturbances [17]. The time dependent wavelength of the ICL can be described as [17,18,19]:

(3) $$v_{1}(t)=v_{0}(t)+A\cos(\omega t)$$

where $v_{0}(t)$ is the central frequency of emitting light, which is determined by the low-frequency component of driving signal, and *A* and *ω* are the amplitude and frequency of the high-frequency component of the driving signal, respectively. By substituting Formula (3) into Formula (1) and expanding it in the form of cosine Fourier series:

(4) $$v_{1}(t)=v_{0}(t)+A\cos(\omega t)$$

where *A_n_* is the amplitude of each harmonic component and can be expressed as follows [20]:

(5) $$A_{n}(v_{0})=\frac{I_{0}\times 2^{1-n}\times C\times L}{n!}\times A^{n}\times \frac{d^{n}\alpha }{dv^{n}}|\begin{array}{c} v=v_{0} \end{array}$$

According to Formula (5), the amplitude of the first harmonic component is:

(6) $$A_{1}(\nu_{0})=I_{0}LA\frac{d\alpha }{d\nu }{|}_{\nu =\nu_{0}}$$

The amplitude of the second harmonic component is:

(7) $$A_{2}(v_{0})=\frac{I_{0}CL}{4}A^{2}\frac{d^{2}\alpha }{dv^{2}}|\begin{array}{c} v=v_{0} \end{array}$$

Based on the above formulas, the amplitudes of the odd harmonic components at the center frequency were 0, and the even harmonic components at the center frequency reached maximum values, which were positively proportional to the gas concentration. As the order increased, the amplitude decreased gradually. In summary, TDLAS-WMS is the optimum choice to analyze the measured gas concentration, which can effectively reduce the 1/*f* noise, increase the signal-to-noise ratio, and improve the MDL performance of the sensor [17].

## 3. System Configuration

**Figure 2:**
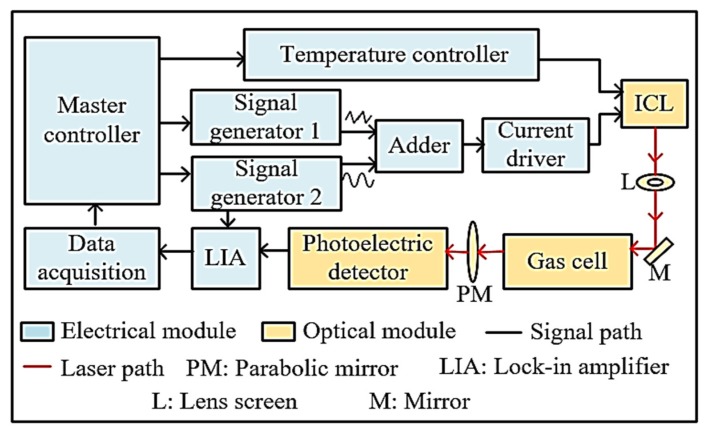
Schematic diagram of the C_2_H_2_ detector.

The ICL laser produced by Nanoplus Co., Gerbrunn, Germany, was used as the light source. Its output wavelength is in the range of 3023 nm to 3027 nm, and the central wavelength is 3025 nm. The embedded thermoelectric cooler Peltier was combined with negative temperature feedback control to guarantee the stability of working temperature of ICL during operation. In terms of the multi-reflection gas cell, the physical length was 40 cm with a volume of 500 mL. The laser was reflected 52 times in the cell, and the effective optical length was increased by up to 20 m. The photodetector is a mid-infrared photoelectric detector: VL5T0 produced by Thorlabs. The detector has good linearity in the spectrum range of 2.7 µm to 4.5 µm, and the response time is less than 120 ns. In addition, the Signal Recovery 7280 lock-in amplifier (LIA) was used to demodulate the second harmonic signal. The trace C_2_H_2_ detector was mainly divided into two modules: electrical and optical. The overall schematic diagram is shown in Figure 2.

The master controller controls signal generator 1, which generates a sinusoidal wave signal with 5 kHz frequency and 0.026 V amplitude, and signal generator 2, which generates a triangular wave signal with 0.5 Hz frequency and 0.2 V amplitude. A high-frequency sinusoidal signal is transmitted to the phase lock-in amplifier as a reference signal. Besides, it is added with a low-frequency triangular wave signal to drive the ICL laser. The emitting light of the ICL with a center wavelength of 3026 nm is tuned by the driving current and the working temperature, which converges through the aperture (L) and goes into the gas cell reflected by the lens (M). Through the measured C_2_H_2_ gas, the output beam is transformed into an electric signal by a photoelectric detector, and then transmitted to the phase locked amplifier. Signal generator 2 provides the phase-locked amplifier with a synchronous signal to ensure phase synchronization. At the output terminal of the phase-locked amplifier, the second harmonic signal can be obtained. Finally, the data acquisition unit processes the measured gas concentration.

## 4. Experiment

### 4.1. Response

To observe the working performance of the trace C_2_H_2_ gas detector, five C_2_H_2_ gases with different standard concentrations (20, 40, 60, 80, and 100 ppbv) were prepared using a dynamic gas dilution equipment. The prepared C_2_H_2_ gases were pumped into the gas cell in sequence at 5 min intervals, and the corresponding peak voltages of the second harmonic signal were obtained and denoted as *max*(2*f*).

**Figure 3:**
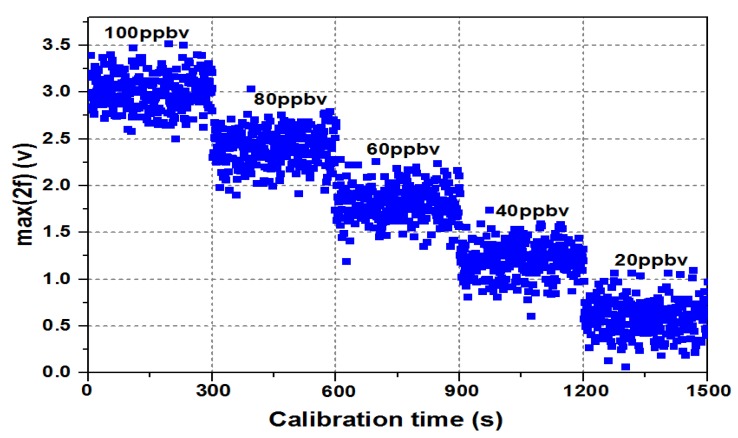
*Max*(2*f*) at different concentrations.

As shown in Figure 3, the x coordinate is the measured time, and the y coordinate is the peak of the second harmonic signal. By analyzing the absorption of the emitting light power from the C_2_H_2_ gas, the peak of the second harmonic signal was linearly decreased by the C_2_H_2_ gas concentration. As a result, this peak was used to represent the C_2_H_2_ gas concentration.

Nevertheless, due to fluctuations of ICL output power, the value of *max*(2*f*) changed slowly over a long observation time (longer than 1 h), and the measured results of different concentrations showed the same growth trend. Seen from Formulas (6) and (7), the laser-induced intensity *I*_0_ as a critical factor of system long-term drift is contained in both of them. The ratio of the second harmonic component to first harmonic component, named as the 2*f*/1*f*-WMS technique, can be utilized to reduce the impact caused by the ICL output power fluctuations [21].

### 4.2. Precision

**Figure 4:**
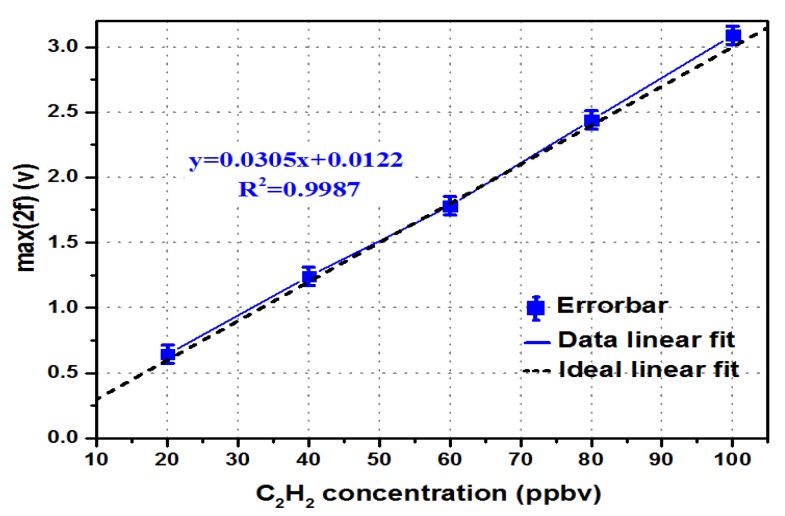
Relationship curves of *max*(2*f*) between the standard concentration and the measured concentration.

Detection precision is a critical parameter for evaluating the sensor performance. The relationship curves of *max*(2*f*) between standard concentration and the measured concentration are shown in Figure 4.

As shown in Figure 4, the x coordinate is the standard concentration of C_2_H_2_, and the y coordinate is the measured peak value of the second harmonic signal. The mean value of each data is expressed in the form of error bars, and the solid blue line is the relationship between the average voltage value of the measured data and the concentration of C_2_H_2_ gas. The black dotted line shows the relationship between the C_2_H_2_ gas concentration and the peak value of the second harmonic signal under theoretical conditions. The results showed that the maximum deviation of the measured data is 0.0412 V, and that the accuracy is 0.4%.

Formula (8) can be obtained by linearly fitting the measured data:

*max*(2*f*) = 0.0305*C* + 0.0122 (8)

where *C* (parts-per-billion volume, ppbv) is the concentration of C_2_H_2_, and then:

*C* = 32.7869 × *max*(2*f*) − 0.4 (9)

Formula (9) can be used to convert the measured peak value of the second harmonic signal into the corresponding C_2_H_2_ gas concentration.

### 4.3. Stability

**Figure 5:**
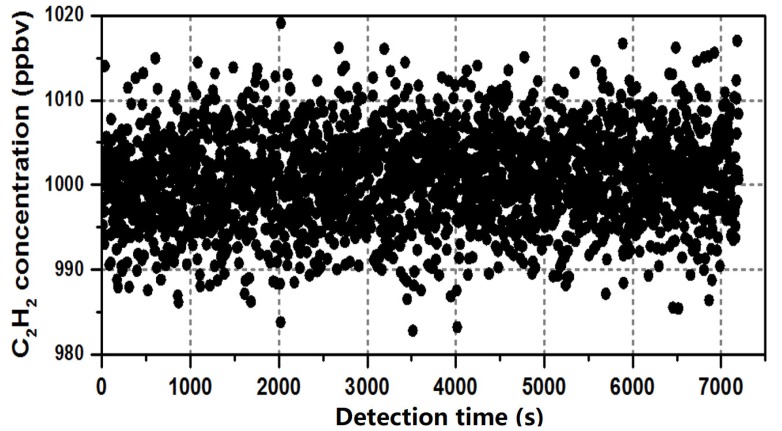
Detection results of 1 ppmv C_2_H_2_ gas concentration in 2 h.

The stability deals with the degree to which sensor characteristics remain constant over time, which is determined by computing the ratio of the maximum deviation and the mean value for a long time of observation at a specific concentration of C_2_H_2_ gas [22]. At room temperature, the C_2_H_2_ gas with a concentration of 1 ppmv was observed for 2 h, the relationship between the gas concentrations was detected using the proposed sensor, and the detection time was recorded. The results are shown in Figure 5.

The x coordinate is the detection time, and the y coordinate is the measured concentration. During the 2 h experimental observation, the peak value of the second harmonic signal ranged from 980 ppbv to 1020 ppbv, and more than 90% of the results were in the range of 990 ppbv to 1010 ppbv with ± 10 ppbv fluctuation. The mean of the measured results was 1000.52 ppbv, and the maximum deviation of the actual data was 17.76 ppbv. Thus, the stability was better than 1.776 × 10^−2^.

In addition, the experimental results changed slowly with the ppbv level during the 2 h observation because of the drift noise of the proposed sensor, indicating that the measurement precision was a critical factor in long-term observation. A reference cell fully filled with pure nitrogen could be utilized to suppress the long-term common noise in future work.

### 4.4. MDL

**Figure 6:**
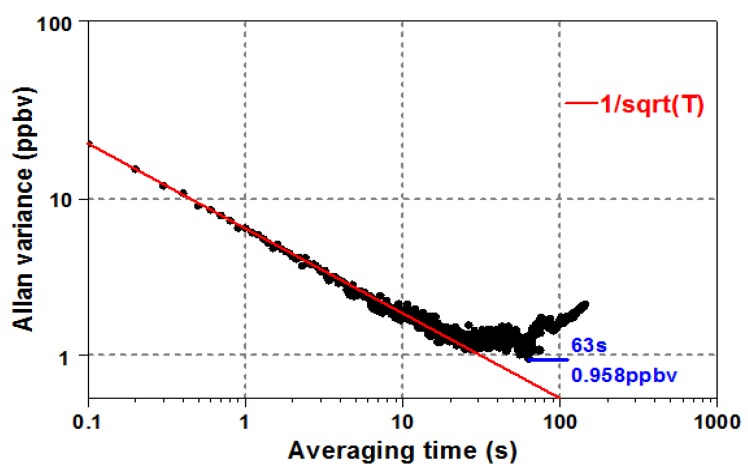
Allan variance of 1 ppmv C_2_H_2_.

As the measured output data drift with time when detecting gas concentration, Allan variance [23] was used to evaluate the experimental data of 1 ppmv C_2_H_2_, as shown in Figure 6.

The results were carried out under laboratory conditions, and the system sampling rate was 10 Hz. As illustrated in Figure 6, the obtained MDL of the proposed sensor was 30 ppbv, with an integration time of 0.1 s. The results of Allan variance analysis showed an appropriate integration time of 63 s, corresponding to an MDL of ~0.958 ppbv. In addition, white noise, one of the main sensor noises, is a random signal having equal intensity at different frequencies. The decreasing red solid line, which is about –1/2, indicates that the theoretical expected behavior of a system is dominated by white noise (before 63 s) [21]. The MDL began to increase after an integration time of 63 s, because the system drift noise dominated in this area.

### 4.5. Recovery Time and Reproducibility

**Figure 7:**
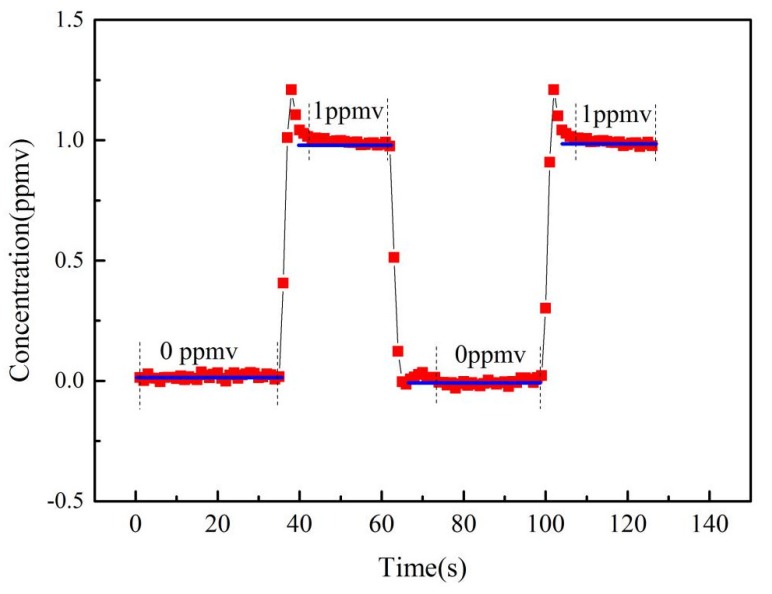
Dynamic measured results of C_2_H_2_ sensor.

Two C_2_H_2_ samples with different concentration levels of 0 ppmv (Pure Nitrogen 99.999%) and 1 ppmv, generated by dynamic gas dilution equipment, were measured to test the performance of the recovery time and reproducibility of the proposed sensor, and the dynamic measured results are shown in Figure 7.

The total measurement time is 130 s under a pressure condition of 760 torr. To test the reproducibility performance, the C_2_H_2_ concentration was initially changed from 0 ppmv to 1 ppmv, then it decreased to 0 ppmv, and finally increases to 1 ppmv. The recovery time includes the gas distribution time, and the process of gas preparation is related to PID control algorithm utilized by dynamic gas dilution equipment. The recovery time for gas sample preparation from a low concentration to high concentration is longer than from high concentration to low concentration, 10 s (0 to 1 ppmv), 15 s (1 to 0 ppmv).

### 4.6. Performance Comparison

In recent years, many researchers have conducted in-depth studies on the detection of C_2_H_2_ gas. The C_2_H_2_ sensor developed in this study is compared with the reported C_2_H_2_ sensors, as shown in Table 1.

Both sensors in [24,25] and the LGA-4500 C_2_H_2_ sensor can detect the gas concentration using the near-infrared band, where the absorption intensity of C_2_H_2_ ranges from 10^−21^ to 10^−20^. Because of the absorption of C_2_H_2_ in the near-infrared band, which is three orders of magnitude weaker than that in the mid-infrared band, the MDL of C_2_H_2_ sensor using absorption line in the near-infrared band remains at the order of ppmv. In addition, because of the use of traditional direct absorption spectroscopy (DAS) in [24], the MDL is still far behind that of the LGA-4500 sensor, although they both use the same absorption band. Therefore, TDLAS-WMS has better MDL performance compared with traditional DAS. In this study, the proposed C_2_H_2_ sensor uses strong absorption line in the mid-infrared band and high-sensitivity TDLAS to obtain superior MDL.

To improve the poor MDL caused by weak absorption intensity in the near-infrared band, one of the most common cavity-enhanced absorption techniques, cavity ring-down spectroscopy (CRDS), is utilized [26]. The MDL of 340 pptv is achieved by employing an external optical cavity with high-reflectivity mirrors as a sample cell. Compared with TDLAS-WMS, CRDS has some disadvantages. First, spectra data cannot be acquired rapidly due to the monochromatic laser source, and the response time is usually at the minute level. Second, analysis is limited by the availability of the tunable laser light at the appropriate wavelength and also at the availability of high-reflectivity mirrors at these wavelengths. Finally, the requirement for laser systems and high-reflectivity mirrors often makes CRDS more expensive than TDLAS-WMS.

Another different spectroscopic method has been developed to obtain gas concentrations with high sensitivity: cavity-enhanced absorption spectroscopy (CEAS). Taking advantage of the high spatial coherence and high brightness of the broadband supercontinuum source, methane and C_2_H_2_ are detected using a mid-infrared spectrum over a bandwidth as large as 450 nm [27]. A MDL of 0.5 ppm for C_2_H_2_ and 0.25 ppm for methane is measured simultaneously, according to the linear response function. Although this gas sensor prototype can retrieve gas concentrations with sub-ppm levels, the MDL and measurement speed of the CEAS technique should be improved in further studies. First, the power spectral density of the mid-infrared source coupled into the chamber can be increased by reducing the connection losses between the light source and the fiber. Second, the measurement speed is currently limited to 30 nm/min, due to monochromator scanning and the long integration time that is needed to improve the signal-to-noise ratio. Although this could be significantly reduced by using a spectrometer with a detector array, a detector with high sensitivity is generally required, which makes this technique more expensive and impractical.

## 5. Conclusions

A trace C_2_H_2_ gas sensor was developed using TDLAS-WMS with an absorption spectrum line at 3305 cm^−1^ (3026 nm). The sensor included an ICL laser, a gas chamber with a 20 m-long optical path, a photodetector, and a phase-locked amplifier. The detection results of the C_2_H_2_ gas with five different concentrations showed a good linear relationship between the peak value of the second harmonic signal and the gas concentration, with a linearity of 0.9987 and a detection accuracy of 0.4%. In total, 1 ppmv of C_2_H_2_ gas sample was used for a 2 h observation, and the measured data show the MDL is as low as 1 ppbv at an integration time of 63 s. In addition, the sensor can be realized by changing the wavelength of the laser to detect a variety of gases, demonstrating flexibility and practicability.

Although the proposed sensor achieved ppbv scales of MDL, it was too large to be suitable and convenient for some applications, such as field measurements (e.g., mobile and airborne). Our new motivation is to develop a gas sensor that is compact and rugged, because mechanical fiber coupling of the diode lasers did not need to be adjusted over several months. Fiber delivery and a fiber beam-coupler will be utilized in our future design, which can reduce the size and thus the ease of operation.

## Figures and Tables

**Table 1:** Performance comparison of the proposed C_2_H_2_ sensor and the reported C_2_H_2_ sensors.

Ref/Type	Wavelength/Maximum Intensity	Technique	MDL (ppmv)	Error (%)
[24]	1.533 µm/1.211 × 10^−20^	DAS	1.8	4
[25]	1.534 nm/8.572 × 10^−21^	TDLAS-WMS	2	1
LGA-4500	1.533 µm/1.211 × 10^−20^	TDLAS-WMS	0.1	1
[26]	1.523 µm/3.145 × 10^−20^	CRDS	0.00034	/
[27]	Broad mid-infrared range	CEAS	0.5	/
This study	1.533 µm/1.211 × 10^−20^	TDLAS-WMS	0.001	0.4
